# Relative Full Completeness for Bicategorical Cartesian Closed Structure

**DOI:** 10.1007/978-3-030-45231-5_15

**Published:** 2020-04-17

**Authors:** Marcelo Fiore, Philip Saville

**Affiliations:** 8 ENS/CNRS, Université Paris-Saclay, Cachan, France; 9 University of Duisburg-Essen, Duisburg, Germany; 10 Department of Computer Science and Technology, University of Cambridge, Cambridge, UK; 11 School of Informatics, University of Edinburgh, Cambridge, UK

**Keywords:** glueing, bicategories, cartesian closure, relative full completeness, rewriting, type theory, conservative extension

## Abstract

The glueing construction, defined as a certain comma category, is an important tool for reasoning about type theories, logics, and programming languages. Here we extend the construction to accommodate ‘2-dimensional theories’ of types, terms between types, and rewrites between terms. Taking bicategories as the semantic framework for such systems, we define the glueing bicategory and establish a bicategorical version of the well-known construction of cartesian closed structure on a glueing category. As an application, we show that free finite-product bicategories are fully complete relative to free cartesian closed bicategories, thereby establishing that the higher-order equational theory of rewriting in the simply-typed lambda calculus is a conservative extension of the algebraic equational theory of rewriting in the fragment with finite products only.

## 1 Introduction

***Relative full completeness for cartesian closed structure.*** Every small category $\mathbb{C}$ can be viewed as an algebraic theory. This has sorts the objects of $\mathbb{C}$ with unary operators for each morphism of $\mathbb{C}$ and equations determined by the equalities in $\mathbb{C}$. Suppose one freely extends $\mathbb{C}$ with finite products. Categorically, one obtains the free cartesian category $\mathbb{F}^{\times}[\mathbb{C}]$ on $\mathbb{C}$. From the well-known construction of $\mathbb{F}^{\times}[\mathbb{C}]$ (see *e.g.* [12] and [46, §8]) it is direct that the universal functor $\mathbb{C} \to \mathbb{F}^{\times}[\mathbb{C}]$ is fully-faithful, a property we will refer to as the *relative full completeness* (*c.f.* [2,16]) of $\mathbb{C}$ in $\mathbb{F}^{\times}[\mathbb{C}]$. Type theoretically, $\mathbb{F}^{\times}[\mathbb{C}]$ corresponds to the Simply-Typed Product Calculus (STPC) over the algebraic theory of $\mathbb{C}$, given by taking the fragment of the Simply-Typed Lambda Calculus (STLC) consisting of just the types, rules, and equational theory for products. Relative full completeness corresponds to the STPC being a *conservative extension*.

Consider now the free cartesian closed category $\mathbb{F}^{\times,\to}[\mathbb{C}]$ on $\mathbb{C}$, type-theoretically corresponding to the STLC over the algebraic theory of $\mathbb{C}$. Does the relative full completeness property, and hence conservativity, still hold for either $\mathbb{C}$ in $\mathbb{F}^{\times,\to}[\mathbb{C}]$

or for $\mathbb{F}^{\times}[\mathbb{C}]$ in $\mathbb{F}^{\times,\to}[\mathbb{C}]$? Precisely, is either the universal functor $\mathbb{C} \to \mathbb{F}^{\times,\to}[\mathbb{C}]$ or its universal cartesian extension $\mathbb{F}^{\times}[\mathbb{C}] \to \mathbb{F}^{\times,\to}[\mathbb{C}]$ full and faithful? The answer is affirmative, but the proof is non-trivial. One must either reason proof-theoretically (*e.g.* in the style of [63, Chapter 8]) or employ semantic techniques such as glueing [39, Annexe C].

In this paper we consider the question of relative full completeness in the bicategorical setting. This corresponds to the question of conservativity for 2-dimensional theories of types, terms between types, and rewrites between terms (see [32,20]). We focus on the particular case of the STLC with invertible rewrites given by $\beta$-reductions and $\eta$-expansions, and its STPC fragment. By identifying these two systems with cartesian closed, resp. finite product, structure 'up to isomorphism' one recovers a conservative extension result for rewrites akin to that for terms.

***2-dimensional categories and rewriting.*** It has been known since the 1980s that one may consider 2-dimensional categories as abstract reduction systems (*e.g.* [54,51]): if sorts are 0-cells (objects) and terms are 1-cells (morphisms), then rewrites between terms ought to be 2-cells. Indeed, every sesquicategory (of which 2-categories are a special class) generates a rewriting relation $\rightsquigarrow$ on its 1-cells defined by $f \rightsquigarrow g$ if and only if there exists a 2-cell $f \Rightarrow g$ (*e.g.* [60,58]). Invertible 2-cells may be then thought of as equality witnesses.

The rewriting rules of the STLC arise naturally in this framework: Seely [56] observed that $\beta$-reduction and $\eta$-expansion may be respectively interpreted as the counit and unit of the adjunctions corresponding to lax (directed) products and exponentials in a 2-category (*c.f.* also [34,27]). This approach was taken up by Hilken [32], who developed a '2-dimensional $\lambda$-calculus' with strict products and lax exponentials to study the proof theory of rewriting in the STLC (*c.f.* also [33]).

Our concern here is with equational theories of rewriting, and we follow Seely in viewing weak categorical structure as a semantic model of rewriting modulo an equational theory. We are not aware of non-syntactic examples of 2-dimensional cartesian closed structure that are lax but not pseudo (*i.e.* up to isomorphism) and so adopt *cartesian closed bicategories* as our semantic framework.

From the perspective of rewriting, a sesquicategory embodies the rewriting of terms modulo the monoid laws for identities and composition, while a bicategory embodies the rewriting of terms modulo the equational theory on rewrites given by the triangle and pentagon laws of a monoidal category. Cartesian closed bicategories further embody the usual $\beta$-reductions and $\eta$-expansions of STLC modulo an equational theory on rewrites; for instance, this identifies the composite rewrite $\langle t_1, t_2\rangle \Rightarrow \langle \pi_1(\langle t_1, t_2\rangle), \pi_2(\langle t_1, t_2\rangle)\rangle \Rightarrow \langle t_1, t_2\rangle$ with the identity rewrite. Indeed, in the free cartesian closed bicategory over a signature of base types and constant terms, the quotient of 1-cells by the isomorphism relation provided by 2-cells is in bijection with $\alpha\beta\eta$-equivalence classes of STLC-terms (*c.f.* [55, Chapter 5]).

***Bicategorical relative full completeness.*** The bicategorical notion of relative full completeness arises by generalising from functors that are fully-faithful to pseudofunctors $F : \mathcal{B} \to \mathcal{C}$ that are *locally an equivalence*, that is, for which every hom-functor $F_{X,Y} : \mathcal{B}(X,Y) \to \mathcal{C}(FX,FY)$ is an equivalence of categories. Interpreted in the context of rewriting, this amounts to the *conservativity of rewriting theories*. First, the equational theory of rewriting in $\mathcal{C}$ is conservative over that in $\mathcal{B}$: the hom-functors do not identify distinct rewrites. Second, the reduction relation in $\mathcal{C}(FX,FY)$ is conservative over that in $\mathcal{B}(X,Y)$: whenever $Ff \rightsquigarrow Fg$ in $\mathcal{C}$ then already $f \rightsquigarrow g$ in $\mathcal{B}$. Third, the term structure in $\mathcal{B}$ gets copied by $F$ in $\mathcal{C}$: modulo the equational theory of rewrites, there are no new terms between types in the image of $F$.

***Contributions.*** This paper makes two main contributions.

Our first contribution, in Section 3, is to introduce the *bicategorical glueing* construction and, in Section 4, to initiate the development of its theory. As well as providing an assurance that our notion is the right one, this establishes the basic framework for applications. Importantly, we bicategorify the fundamental folklore result (*e.g.* [40,12,62]) establishing mild conditions under which a glued bicategory is cartesian closed.

Our second contribution, in Section 5, is to employ bicategorical glueing to show that for a bicategory $\mathcal{B}$ with finite-product completion $\mathcal{F}^{\times}[\mathcal{B}]$ and cartesian-closed completion $\mathcal{F}^{\times,\to}[\mathcal{B}]$, the universal pseudofunctor $\mathcal{B} \to \mathcal{F}^{\times,\to}[\mathcal{B}]$ and its universal finite-product-preserving extension $\mathcal{F}^{\times}[\mathcal{B}] \to \mathcal{F}^{\times,\to}[\mathcal{B}]$ are both locally an equivalence. Since one may directly observe that the universal pseudofunctor $\mathcal{B} \to \mathcal{F}^{\times}[\mathcal{B}]$ is locally an equivalence, we obtain *relative full completeness results for bicategorical cartesian closed structure* mirroring those of the categorical setting. Establishing this proof-theoretically would require the development of a 2-dimensional proof theory. Given the complexities already present at the categorical level this seems a serious and interesting undertaking. Here, once the basic bicategorical theory has been established, the proof is relatively compact. This highlights the effectiveness of our approach for the application.

The result may also be expressed type-theoretically. For instance, in terms of the type theories of [20], the type theory $\Lambda_{\text{ps}}^{\times,\to}$ for cartesian closed bicategories is a conservative extension of the type theory $\Lambda_{\text{ps}}^{\times}$ for finite-product bicategories. It follows that, modulo the equational theory of bicategorical products and exponentials, any rewrite between STPC-terms constructed using the $\beta\eta$-rewrites for both products and exponentials may be equally presented as constructed from just the $\beta\eta$-rewrites for products (see [21,55]).

***Further work.*** We view the foundational theory presented here as the starting point for future work. For instance, we plan to incorporate further type structure into the development, such as coproducts (*c.f.* [22,16,4]) and monoidal structure (*c.f.* [31]).

On the other hand, the importance of glueing in the categorical setting suggests that its bicategorical counterpart will find a range of applications. A case in point, which has already been developed, is the proof of a 2-dimensional normalisation property for the type theory $\Lambda_{\text{ps}}^{\times,\to}$ for cartesian closed bicategories of [20] that entails a corresponding bicategorical coherence theorem [21,55]. There are also a variety of syntactic constructions in programming languages and type theory that naturally come with a 2-dimensional semantics (see *e.g.* the use of 2-categorical constructions in [23,14,6,61,35]). In such scenarios, bicategorical glueing may prove useful for establishing properties corresponding to the notions of adequacy and/or canonicity, or for proving further conservativity properties.

## 2 Cartesian closed bicategories

We begin by briefly recapitulating the basic theory of bicategories, including the definition of cartesian closure. A summary of the key definitions is in [41]; for a more extensive introduction see *e.g.* [5,7].

### 2.1 Bicategories

Bicategories axiomatise structures in which the associativity and unit laws of composition only hold up to coherent isomorphism, for instance when composition is defined by a universal property. They are rife in mathematics and theoretical computer science, appearing in the semantics of computation [29,11,49], datatype models [1,13], categorical logic [26], and categorical algebra [19,25,18].

**Definition 1 ([5]).** *A* bicategory $\mathcal{B}$ *consists of*

1. *A class of objects* $ob(\mathcal{B})$*,*
2. *For every* $X, Y \in ob(\mathcal{B})$ *a* hom-category $\big(\mathcal{B}(X,Y), \bullet, \mathrm{id}\big)$ *with objects* 1-cells $f : X \to Y$ *and morphisms* 2-cells $\alpha : f \Rightarrow f' : X \to Y$*; composition of 2-cells is called* vertical composition*,*
3. *For every* $X, Y, Z \in ob(\mathcal{B})$ *an* identity *functor* $\mathrm{Id}_X : \mathbf{1} \to \mathcal{B}(X,X)$ *(for* $\mathbf{1}$ *the terminal category) and a* horizontal composition *functor* $\circ_{X,Y,Z} : \mathcal{B}(Y,Z) \times \mathcal{B}(X,Y) \to \mathcal{B}(X,Z)$*,*
4. *Invertible 2-cells*

$$\begin{aligned} \mathbf{a}_{h,g,f} &: (h \circ g) \circ f \Rightarrow h \circ (g \circ f) : W \to Z \\ \mathbf{l}_f &: \mathrm{Id}_X \circ f \Rightarrow f : W \to X \\ \mathbf{r}_g &: g \circ \mathrm{Id}_X \Rightarrow g : X \to Y \end{aligned}$$

*for every* $f : W \to X$*,* $g : X \to Y$ *and* $h : Y \to Z$*, natural in each of their parameters and satisfying a* triangle law *and a* pentagon law *analogous to those for monoidal categories.*

*A bicategory is said to be* locally small *if every hom-category is small.*

*Example 1.* 1. Every 2-category is a bicategory in which the structural isomorphisms are all the identity.
2. For any category $\mathbb{C}$ with pullbacks there exists a *bicategory of spans* over $\mathbb{C}$ [5]. The objects are those of $\mathbb{C}$, 1-cells $A \rightsquigarrow B$ are spans $(A \leftarrow X \to B)$, and 2-cells $(A \leftarrow X \to B) \to (A \leftarrow X' \to B)$ are morphisms $X \to X'$ making the expected diagram commute. Composition is defined using chosen pullbacks.

A bicategory has three notions of 'opposite', depending on whether one reverses 1-cells, 2-cells, or both (see *e.g.* [37, §1.6]). We shall only require the following.

**Definition 2.** *The* opposite *of a bicategory $\mathcal{B}$, denoted $\mathcal{B}^{\mathrm{op}}$, is obtained by setting $\mathcal{B}^{\mathrm{op}}(X,Y) := \mathcal{B}(Y,X)$ for all $X, Y \in \mathcal{B}$.*

A morphism of bicategories is called a *pseudofunctor* (or *homomorphism*) [5]. It is a mapping on objects, 1-cells and 2-cells that preserves horizontal composition up to isomorphism. Vertical composition is preserved strictly.

**Definition 3.** *A* pseudofunctor $(F, \phi, \psi) : \mathcal{B} \to \mathcal{C}$ *between bicategories $\mathcal{B}$ and $\mathcal{C}$ consists of*

1. *A mapping $F : ob(\mathcal{B}) \to ob(\mathcal{C})$,*
2. *A functor $F_{X,Y} : \mathcal{B}(X,Y) \to \mathcal{C}(FX, FY)$ for every $X, Y \in ob(\mathcal{B})$,*
3. *An invertible 2-cell $\psi_X : \mathrm{Id}_{FX} \Rightarrow F(\mathrm{Id}_X)$ for every $X \in ob(\mathcal{B})$,*
4. *An invertible 2-cell $\phi_{f,g} : F(f) \circ F(g) \Rightarrow F(f \circ g)$ for every $g : X \to Y$ and $f : Y \to Z$, natural in $f$ and $g$,*

*subject to two unit laws and an associativity law. A pseudofunctor for which $\phi$ and $\psi$ are both the identity is called* strict*. A pseudofunctor is called* locally $P$ *if every functor $F_{X,Y}$ satisfies the property $P$.*

*Example 2.* A monoidal category is equivalently a one-object bicategory; a monoidal functor is equivalently a pseudofunctor between one-object bicategories.

Pseudofunctors $F, G : \mathcal{B} \to \mathcal{C}$ are related by *pseudonatural transformations*. A pseudonatural transformation $(\mathsf{k}, \overline{\mathsf{k}}) : F \Rightarrow G$ consists of a family of 1-cells $(\mathsf{k}_X : FX \to GX)_{X \in \mathcal{B}}$ and, for every $f : X \to Y$, an invertible 2-cell $\overline{\mathsf{k}}_f : \mathsf{k}_Y \circ Ff \Rightarrow Gf \circ \mathsf{k}_X$ witnessing naturality. The 2-cells $\overline{\mathsf{k}}_f$ are required to be natural in $f$ and satisfy two coherence axioms. A morphism of pseudonatural transformations is called a *modification*, and may be thought of as a coherent family of 2-cells.

*Notation 1.* For bicategories $\mathcal{B}$ and $\mathcal{C}$ we write $\mathbf{Bicat}(\mathcal{B}, \mathcal{C})$ for the (possibly large) bicategory of pseudofunctors, pseudonatural transformations, and modifications (see *e.g.* [41]). If $\mathcal{C}$ is a 2-category, then so is $\mathbf{Bicat}(\mathcal{B}, \mathcal{C})$. We write $\mathbf{Cat}$ for the 2-category of small categories and think of the 2-category $\mathbf{Bicat}(\mathcal{B}^{\mathrm{op}}, \mathbf{Cat})$ as a bicategorical version of the presheaf category $\mathrm{Set}^{\mathbb{C}^{\mathrm{op}}}$. As for presheaf categories, one must take care to avoid size issues. We therefore adopt the convention that when considering $\mathbf{Bicat}(\mathcal{B}^{\mathrm{op}}, \mathbf{Cat})$ the bicategory $\mathcal{B}$ is small or locally small as appropriate.

*Example 3.* For every bicategory $\mathcal{B}$ and $X \in \mathcal{B}$ there exists the *representable pseudofunctor* $\mathrm{Y}X : \mathcal{B}^{\mathrm{op}} \to \mathbf{Cat}$, defined by $\mathrm{Y}X := \mathcal{B}(-, X)$. The 2-cells $\phi$ and $\psi$ are structural isomorphisms.

The notion of equivalence between bicategories is called biequivalence. A *biequivalence* $\mathcal{B} \simeq \mathcal{C}$ consists of a pair of pseudofunctors $F : \mathcal{B} \leftrightarrows G : \mathcal{C}$ together with equivalences $FG \simeq \mathrm{id}_{\mathcal{C}}$ and $GF \simeq \mathrm{id}_{\mathcal{B}}$ in $\mathbf{Bicat}(\mathcal{C}, \mathcal{C})$ and $\mathbf{Bicat}(\mathcal{B}, \mathcal{B})$ respectively. Equivalences in an arbitrary bicategory are defined by analogy with equivalences of categories, see *e.g.* [42, pp. 28].

*Remark 1.* The coherence theorem for monoidal categories [44, Chapter VII] generalises to bicategories: any bicategory is biequivalent to a 2-category [45] (see [42] for a readable summary of the argument). We are therefore justified in writing simply $\cong$ for composites of $\mathbf{a}, \mathbf{l}$ and $\mathbf{r}$.

As a rule of thumb, a category-theoretic proposition lifts to a bicategorical proposition so long as one takes care to weaken isomorphisms to equivalences and sprinkle the prefixes 'pseudo' and 'bi' in appropriate places. For instance, bicategorical adjoints are called *biadjoints* and bicategorical limits are called *bilimits* [59]. The latter may be thought of as limits in which every cone is filled by a coherent choice of invertible 2-cell. Bilimits are preserved by representable pseudofunctors and by right biadjoints. The *bicategorical Yoneda lemma* [59, §1.9] says that for any pseudofunctor $P : \mathcal{B}^{\mathrm{op}} \to \mathbf{Cat}$, evaluation at the identity determines a pseudonatural family of equivalences $\mathbf{Bicat}(\mathcal{B}^{\mathrm{op}}, \mathbf{Cat})(\mathrm{Y}X, P) \simeq PX$. One may then deduce that the *Yoneda pseudofunctor* $\mathrm{Y} : \mathcal{B} \to \mathbf{Bicat}(\mathcal{B}^{\mathrm{op}}, \mathbf{Cat}) : X \mapsto \mathrm{Y}X$ is locally an equivalence. Another 'bicategorified' lemma is the following, which we shall employ in Section 5.

**Lemma 1.** *1. For pseudofunctors $F, G : \mathcal{B} \to \mathcal{C}$, if $F \simeq G$ and $G$ is locally an equivalence, then so is $F$.*
*2. For pseudofunctors $F : \mathcal{A} \to \mathcal{B}$, $G : \mathcal{B} \to \mathcal{C}$, $H : \mathcal{C} \to \mathcal{D}$, if $G \circ F$ and $H \circ G$ are local equivalences, then so is $F$.*

### 2.2 fp-Bicategories

It is convenient to directly consider all finite products, as this reduces the need to deal with the equivalent objects given by re-bracketing binary products. To avoid confusion with the 'cartesian bicategories' of Carboni and Walters [10,8], we call a bicategory with all finite products an *fp-bicategory*.

**Definition 4.** *An* fp-bicategory $(\mathcal{B}, \Pi_n(-))$ *is a bicategory $\mathcal{B}$ equipped with the following data for every $A_1, \ldots, A_n \in \mathcal{B}$ $(n \in \mathbb{N})$:*

1. *A chosen object $\prod_n(A_1, \ldots, A_n)$,*
2. *Chosen arrows $\pi_k : \prod_n(A_1, \ldots, A_n) \to A_k$ $(k = 1, \ldots, n)$, called* projections*,*
3. *For every $X \in \mathcal{B}$ an adjoint equivalence*

$$\mathcal{B}\left(X, \textstyle\prod_n(A_1, \ldots, A_n)\right) \underset{\langle -, \ldots, = \rangle}{\overset{(\pi_1 \circ -, \ldots, \pi_n \circ -)}{\perp\simeq}} \textstyle\prod_{i=1}^{n} \mathcal{B}(X, A_i) \tag{1}$$

*specified by choosing a family of universal arrows (see* e.g. *[44, Theorem IV.2]) with components $\varpi^{(i)}_{f_1, \ldots, f_n} : \pi_i \circ \langle f_1, \ldots, f_n \rangle \Rightarrow f_i$ for $i = 1, \ldots, n$.*

*We call the right adjoint* $\langle -, \ldots, = \rangle$ *the* $n$-ary tupling.

Explicitly, the universal property of $\varpi = (\varpi^{(1)}, \ldots, \varpi^{(n)})$ is the following. For any finite family of 2-cells $(\alpha_i : \pi_i \circ g \Rightarrow f_i : X \to A_i)_{i=1,\ldots,n}$, there exists a 2-cell $\mathsf{p}^\dagger(\alpha_1, \ldots, \alpha_n) : g \Rightarrow \langle f_1, \ldots, f_n \rangle : X \to \prod_n(A_1, \ldots, A_n)$, unique such that

$$\varpi^{(k)}_{f_1,\ldots,f_n} \bullet \big(\pi_k \circ \mathsf{p}^\dagger(\alpha_1, \ldots, \alpha_n)\big) = \alpha_k : \pi_k \circ g \Rightarrow f_k$$

for $k = 1, \ldots, n$. One thereby obtains a functor $\langle -, \ldots, = \rangle$ and an adjunction as in (1) with counit $\varpi = (\varpi^{(1)}, \ldots, \varpi^{(n)})$ and unit $\varsigma_g := \mathsf{p}^\dagger(\mathrm{id}_{\pi_1 \circ g}, \ldots, \mathrm{id}_{\pi_n \circ g}) : g \Rightarrow \langle \pi_1 \circ g, \ldots, \pi_n \circ g \rangle$. This defines a *lax* $n$-ary product structure: one merely obtains an adjunction in (1). One turns it into a bicategorical (*pseudo*) product by further requiring the unit and counit to be invertible. The *terminal object* **1** arises as $\prod_0()$. We adopt the same notation as for categorical products, for example by writing $\prod_{i=1}^n A_i$ for $\prod_n(A_1, \ldots, A_n)$ and $\prod_{i=1}^n f_i$ for $\langle f_1 \circ \pi_1, \ldots, f_n \circ \pi_n \rangle$.

*Example 4.* The bicategory of spans over a *lextensive category* [9] has finite products; such a bicategory is biequivalent to its opposite, so these are in fact biproducts [38, Theorem 6.2]. Biproduct structure arises using the coproduct structure of the underlying category (*c.f.* the biproduct structure of the category of relations).

*Remark 2 (*c.f. *Remark 1).* fp-Bicategories satisfy the following coherence theorem: every fp-bicategory is biequivalent to a 2-category with 2-categorical products [52, Theorem 4.1]. Thus, we shall sometimes simply write $\cong$ in diagrams for composites of 2-cells arising from either the bicategorical or product structure. In pasting diagrams we shall omit such 2-cells completely (*c.f.* [30, Remark 3.1.16]; for a detailed exposition, see [64, Appendix A]).

One may think of bicategorical product structure as an intensional version of the familiar categorical structure, except the usual equations (*e.g.* [28]) are now witnessed by natural families of invertible 2-cells. It is useful to introduce explicit names for these 2-cells.

*Notation 2.* In the following, and throughout, we write $A_\bullet$ for a finite sequence $\langle A_1, \ldots, A_n \rangle$.

**Lemma 2.** *For any fp-bicategory* $(\mathcal{B}, \Pi_n(-))$ *there exist canonical choices for the following natural families of invertible 2-cells:*

1. *For every* $(h_i : Y \to A_i)_{i=1,\ldots,n}$ *and* $g : X \to Y$*, a 2-cell* $\mathsf{post}(h_\bullet; g) : \langle h_1, \ldots, h_n \rangle \circ g \Rightarrow \langle h_1 \circ g, \ldots, h_n \circ g \rangle$*,*
2. *For every* $(h_i : A_i \to B_i)_{i=1,\ldots,n}$ *and* $(g_i : X \to A_i)_{i=1,\ldots,n}$*, a 2-cell* $\mathsf{fuse}(h_\bullet; g_\bullet) : (\prod_{i=1}^n h_i) \circ \langle g_1, \ldots, g_n \rangle \Rightarrow \langle h_1 \circ g_1, \ldots, h_n \circ g_n \rangle$*.*

In particular, it follows from Lemma 2(2) that there exists a canonical natural family of invertible 2-cells $\Phi_{h_\bullet, g_\bullet} : (\prod_{i=1}^n h_i) \circ (\prod_{i=1}^n g_i) \Rightarrow \prod_{i=1}^n (h_i \circ g_i)$ for any $(h_i : A_i \to B_i)_{i=1,\ldots,n}$ and $(g_j : X_j \to A_j)_{j=1,\ldots,n}$.

In the categorical setting, a cartesian functor preserves products up to isomorphism. An fp-pseudofunctor preserves bicategorical products up to equivalence.

**Definition 5.** *An* fp-pseudofunctor $(F, \mathrm{q}^{\times})$ *between fp-bicategories* $(\mathcal{B}, \Pi_n(-))$ *and* $(\mathcal{C}, \Pi_n(-))$ *is a pseudofunctor* $F : \mathcal{B} \to \mathcal{C}$ *equipped with specified equivalences*

$$\langle F\pi_1, \ldots, F\pi_n \rangle : F(\textstyle\prod_{i=1}^{n} A_i) \leftrightarrows \prod_{i=1}^{n}(FA_i) : \mathrm{q}^{\times}_{A_\bullet}$$

*for every* $A_1, \ldots, A_n \in \mathcal{B}$ $(n \in \mathbb{N})$*. We denote the 2-cells witnessing these equivalences by* $\mathsf{u}^{\times}_{A_\bullet} : \mathrm{Id}_{(\prod_i FA_i)} \Rightarrow \langle F\pi_1, \ldots, F\pi_n \rangle \circ \mathrm{q}^{\times}_{A_\bullet}$ *and* $\mathsf{c}^{\times}_{A_\bullet} : \mathrm{q}^{\times}_{A_\bullet} \circ \langle F\pi_1, \ldots, F\pi_n \rangle \Rightarrow \mathrm{Id}_{(F\Pi_i A_i)}$*. We call* $(F, \mathrm{q}^{\times})$ strict *if* $F$ *is strict and satisfies*

$$F(\textstyle\prod_n(A_1, \ldots, A_n)) = \prod_n(FA_1, \ldots, FA_n)$$

$$F(\pi_i^{A_1,\ldots,A_n}) = \pi_i^{FA_1,\ldots,FA_n} \qquad F\varpi^{(i)}_{t_1,\ldots,t_n} = \varpi^{(i)}_{Ft_1,\ldots,Ft_n}$$

$$F\langle t_1, \ldots, t_n \rangle = \langle Ft_1, \ldots, Ft_n \rangle \qquad \mathrm{q}^{\times}_{A_1,\ldots,A_n} = \mathrm{Id}_{\Pi_n(FA_1,\ldots,FA_n)}$$

*with equivalences given by the* 2*-cells* $\mathsf{p}^{\dagger}(\mathbf{r}_{\pi_1}, \ldots, \mathbf{r}_{\pi_n}) : \mathrm{Id} \overset{\cong}{\Rightarrow} \langle \pi_1, \ldots, \pi_n \rangle$*.*

*Notation 3.* For fp-bicategories $\mathcal{B}$ and $\mathcal{C}$ we write $\mathbf{fp\text{-}Bicat}(\mathcal{B}, \mathcal{C})$ for the bicategory of fp-pseudofunctors, pseudonatural transformations and modifications.[3]

We define two further families of 2-cells to witness standard properties of cartesian functors. The first witnesses the fact that any fp-pseudofunctor commutes with the $\prod_n(-, \ldots, =)$ operation. The second witnesses the equality $\langle F\pi_1, \ldots, F\pi_n \rangle \circ F\langle f_1, \ldots, f_n \rangle = \langle Ff_1, \ldots, Ff_n \rangle$ 'unpacking' an $n$-ary tupling from inside $F$.

**Lemma 3.** *Let* $(F, \mathrm{q}^{\times}) : (\mathcal{B}, \Pi_n(-)) \to (\mathcal{C}, \Pi_n(-))$ *be an fp-pseudofunctor.*

1. *For any finite family of 1-cells* $(f_i : A_i \to A'_i)_{i=1,\ldots,n}$ *in* $\mathcal{B}$*, there exists an invertible 2-cell* $\mathsf{nat}_{f_\bullet} : \mathrm{q}^{\times}_{A'_\bullet} \circ \prod_{i=1}^{n} Ff_i \Rightarrow F(\prod_{i=1}^{n} f_i) \circ \mathrm{q}^{\times}_{A_\bullet}$ *such that the pair* $(\mathrm{q}^{\times}, \mathsf{nat})$ *forms a a pseudonatural transformation*
$$\textstyle\prod_{i=1}^{n}(F(-), \ldots, F(=)) \Rightarrow (F \circ \prod_{i=1}^{n})(-, \ldots, =)$$
2. *For any finite family of 1-cells* $(f_i : X \to B_i)_{i=1,\ldots,n}$ *in* $\mathcal{B}$*, there exists a canonical choice of naturally invertible 2-cell* $\mathsf{unpack}_{f_\bullet} : \langle F\pi_1, \ldots, F\pi_n \rangle \circ F\langle f_1, \ldots, f_n \rangle \Rightarrow \langle Ff_1, \ldots, Ff_n \rangle : FX \to \prod_{i=1}^{n} FB_i$*.*

### 2.3 Cartesian closed bicategories

A cartesian closed bicategory is an fp-bicategory $(\mathcal{B}, \Pi_n(-))$ equipped with a biadjunction $(-) \times A \dashv (A \Rightarrow -)$ for every $A \in \mathcal{B}$. Examples include the bicategory of generalised species [17], bicategories of concurrent games [49], and bicategories of operads [26].

[3] In the categorical setting, every natural transformation between cartesian functors is monoidal with respect to the cartesian structure and a similar fact is true bicategorically: every pseudonatural transformation is canonically compatible with the product structure, see [55, § 4.1.1].

**Definition 6.** *A* cartesian closed bicategory *or* cc-bicategory *is an fp-bicategory* $(\mathcal{B}, \Pi_n(-))$ *equipped with the following data for every* $A, B \in \mathcal{B}$*:*

1. *A chosen object* $(A \Rightarrow B)$*,*
2. *A specified 1-cell* $\mathrm{eval}_{A,B} : (A \Rightarrow B) \times A \to B$*,*
3. *For every* $X \in \mathcal{B}$*, an adjoint equivalence*

$$\mathcal{B}(X, A \Rightarrow B) \underset{\lambda}{\overset{\mathrm{eval}_{A,B} \circ (- \times A)}{\rightleftarrows}} \mathcal{B}(X \times A, B) \quad \perp\simeq$$

*specified by a choice of universal arrow* $\varepsilon_f : \mathrm{eval}_{A,B} \circ (\lambda f \times A) \overset{\cong}{\Rightarrow} f$*.*

*We call the functor* $\lambda(-)$ currying *and refer to* $\lambda f$ *as the* currying of $f$*.*

Explicitly, the counit $\varepsilon$ satisfies the following universal property. For every 1-cell $g : X \to (A \Rightarrow B)$ and 2-cell $\alpha : \mathrm{eval}_{A,B} \circ (g \times A) \Rightarrow f$ there exists a unique 2-cell $\mathsf{e}^{\dagger}(\alpha) : g \Rightarrow \lambda f$ such that $\varepsilon_f \bullet \big(\mathrm{eval}_{A,B} \circ (\mathsf{e}^{\dagger}(\alpha) \times A)\big) = \alpha$. This defines a *lax* exponential structure. One obtains a *pseudo* (bicategorical) exponential structure by further requiring that $\varepsilon$ and the unit $\eta_t := \mathsf{e}^{\dagger}(\mathrm{id}_{\mathrm{eval}_{A,B} \circ (t \times A)})$ are invertible.

*Example 5.* Every 'presheaf' 2-category $\mathbf{Bicat}(\mathcal{B}^{\mathrm{op}}, \mathbf{Cat})$ has all bicategorical limits [52, Proposition 3.6], given pointwise, and is cartesian closed with $(P \Rightarrow Q)X := \mathbf{Bicat}(\mathcal{B}^{\mathrm{op}}, \mathbf{Cat})(\mathrm{Y}X \times P, Q)$ [55, Chapter 6].

As for products, we adopt the notational conventions that are standard in the categorical setting, for example by writing $(f \Rightarrow g) : (A \Rightarrow B) \to (A' \Rightarrow B')$ for the currying of $(g \circ \mathrm{eval}_{A,B}) \circ (\mathrm{Id}_{A \Rightarrow B} \times f)$.

Just as fp-pseudofunctors preserve products up to equivalence, cartesian closed pseudofunctors preserve products and exponentials up to equivalence.

**Definition 7.** *A* cartesian closed pseudofunctor *or* cc-pseudofunctor *between cc-bicategories* $(\mathcal{B}, \Pi_n(-), \Rightarrow)$ *and* $(\mathcal{C}, \Pi_n(-), \Rightarrow)$ *is an fp-pseudofunctor* $(F, \mathrm{q}^{\times})$ *equipped with specified equivalences* $m_{A,B} : F(A \Rightarrow B) \leftrightarrows (FA \Rightarrow FB) : \mathrm{q}^{\Rightarrow}_{A,B}$ *for every* $A, B \in \mathcal{B}$*, where* $m_{A,B} : F(A \Rightarrow B) \to (FA \Rightarrow FB)$ *is the currying of* $F(\mathrm{eval}_{A,B}) \circ \mathrm{q}^{\times}_{A \Rightarrow B, A}$*. A cc-pseudofunctor* $(F, \mathrm{q}^{\times}, \mathrm{q}^{\Rightarrow})$ *is* strict *if* $(F, \mathrm{q}^{\times})$ *is a strict fp-pseudofunctor such that*

$$F(A \Rightarrow B) = (FA \Rightarrow FB)$$
$$F(\mathrm{eval}_{A,B}) = \mathrm{eval}_{FA,FB} \qquad F(\varepsilon_t) = \varepsilon_{Ft}$$
$$F(\lambda t) = \lambda(Ft) \qquad \mathrm{q}^{\Rightarrow}_{A,B} = \mathrm{Id}_{FA \Rightarrow FB}$$

*with equivalences given by the* 2*-cells*

$$\mathsf{e}^{\dagger}(\mathrm{eval}_{FA,FB} \circ \kappa) : \mathrm{Id}_{(FA \Rightarrow FB)} \overset{\cong}{\Rightarrow} \lambda(\mathrm{eval}_{FA,FB} \circ \mathrm{Id}_{(FA \Rightarrow FB) \times FA})$$

*where* $\kappa$ *is the canonical isomorphism* $\mathrm{Id}_{FA \Rightarrow FB} \times FA \cong \mathrm{Id}_{(FA \Rightarrow FB) \times FA}$*.*

*Remark 3.* As is well-known in the case of **Cat** (*e.g.* [44, IV.2]), every equivalence $X \simeq Y$ in a bicategory gives rise to an *adjoint equivalence* between $X$ and $Y$ with the same 1-cells (see *e.g.* [42, pp. 28–29]). Thus, one may assume without loss of generality that all the equivalences in the preceding definition are adjoint equivalences. The same observation applies to the definition of fp-pseudofunctors.

*Notation 4.* For cc-bicategories $\mathcal{B}$ and $\mathcal{C}$ we write $\mathbf{cc\text{-}Bicat}(\mathcal{B}, \mathcal{C})$ for the bicategory of cc-pseudofunctors, pseudonatural transformations and modifications (*c.f.* Notation 3).

## 3 Bicategorical glueing

The glueing construction has been discovered in various forms, with correspondingly various names: the notions of logical relation [50,57], sconing [24], Freyd covers, and glueing (*e.g.* [40]) are all closely related (see *e.g.* [47] for an overview of the connections). Originally presented set-theoretically, the technique was quickly given categorical expression [43,47] and is now a standard component of the armoury for studying type theories (*e.g.* [40,12]).

The *glueing* $\mathrm{gl}(F)$ of categories $\mathbb{C}$ and $\mathbb{D}$ along a functor $F : \mathbb{C} \to \mathbb{D}$ may be defined as the comma category $(\mathrm{id}_{\mathbb{D}} \downarrow F)$. We define bicategorical glueing analogously.

**Definition 8.**

1. *Let $F : \mathcal{A} \to \mathcal{C}$ and $G : \mathcal{B} \to \mathcal{C}$ be pseudofunctors of bicategories. The* comma bicategory $(F \downarrow G)$ *has objects triples $(A \in \mathcal{A}, f : FA \to GB, B \in \mathcal{B})$. The 1-cells $(A, f, B) \to (A', f', B')$ are triples $(p, \alpha, q)$, where $p : A \to A'$ and $q : B \to B'$ are 1-cells and $\alpha$ is an invertible 2-cell $\alpha : f' \circ Fp \Rightarrow Gq \circ f$. The 2-cells $(p, \alpha, q) \Rightarrow (p', \alpha', q')$ are pairs of 2-cells $(\sigma : p \Rightarrow p', \tau : q \Rightarrow q')$ such that the following diagram commutes:*

$$\begin{array}{ccc} f' \circ F(p) & \xrightarrow{f' \circ F(\sigma)} & f' \circ F(p') \\ {\scriptstyle \alpha}\downarrow & & \downarrow{\scriptstyle \alpha'} \\ G(q) \circ f & \xrightarrow[G(\tau) \circ f]{} & G(q') \circ f \end{array} \tag{2}$$

*Identities and horizontal composition are given by the following pasting diagrams.*

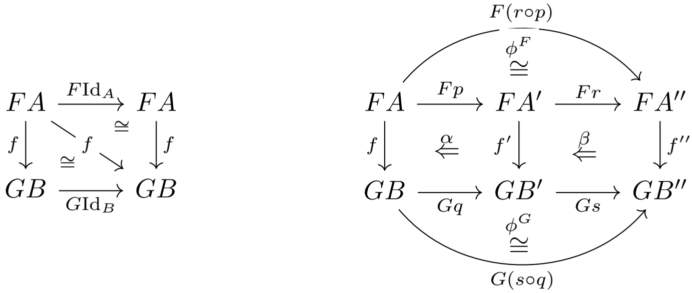

*Vertical composition, the identity 2-cell, and the structural isomorphisms are given component-wise.*

2. *The* glueing bicategory $\mathrm{gl}(\mathfrak{J})$ *of bicategories* $\mathcal{B}$ *and* $\mathcal{C}$ *along a pseudofunctor* $\mathfrak{J} : \mathcal{B} \to \mathcal{C}$ *is the comma bicategory* $(\mathrm{id}_{\mathcal{C}} \downarrow \mathfrak{J})$*.*

We call axiom (2) the *cylinder condition* due to its shape when viewed as a (3-dimensional) pasting diagram. Note that one directly obtains projection pseudofunctors $\mathcal{B} \xleftarrow{\pi_{\mathrm{dom}}} \mathrm{gl}(\mathfrak{J}) \xrightarrow{\pi_{\mathrm{cod}}} \mathcal{C}$.

We develop some basic theory of glueing bicategories, which we shall put to use in Section 5. We follow the terminology of [15].

**Definition 9.** *Let* $\mathfrak{J} : \mathcal{B} \to \mathcal{X}$ *be a pseudofunctor. The* relative hom-pseudofunctor $\langle\mathfrak{J}\rangle : \mathcal{X} \to \mathbf{Bicat}(\mathcal{B}^{\mathrm{op}}, \mathbf{Cat})$ *is defined by* $\langle\mathfrak{J}\rangle X := \mathcal{X}(\mathfrak{J}(-), X)$*.*

Following [15], one might call the glueing bicategory $\mathrm{gl}(\langle\mathfrak{J}\rangle)$ associated to a relative hom-pseudofunctor the bicategory of *$\mathcal{B}$-intensional Kripke relations of arity* $\mathfrak{J}$, and view it as an intensional, bicategorical, version of the category of Kripke relations.

The relative hom-pseudofunctor preserves all bilimits that exist in its domain. For products, this may be described explicitly.

**Lemma 4.** *For any fp-bicategory* $(\mathcal{X}, \Pi_n(-))$ *and pseudofunctor* $\mathfrak{J} : \mathcal{B} \to \mathcal{X}$*, the relative hom-pseudofunctor* $\langle\mathfrak{J}\rangle$ *extends canonically to an fp-pseudofunctor.*

*Proof.* *Take* $\mathrm{q}^{\times}_{X_\bullet}$ *to be the n-ary tupling* $\prod_{i=1}^{n} \mathcal{X}(\mathfrak{J}(-), X_i) \xrightarrow{\simeq} \mathcal{X}(\mathfrak{J}(-), \prod_{i=1}^{n} X_i)$*. This forms a pseudonatural transformation with naturality witnessed by* $\mathsf{post}$*.*

For any pseudofunctor $\mathfrak{J} : \mathcal{B} \to \mathcal{X}$ there exists a pseudonatural transformation $(l, \bar{l}) : \mathrm{Y} \Rightarrow \langle\mathfrak{J}\rangle \circ \mathfrak{J} : \mathcal{B} \to \mathbf{Bicat}(\mathcal{B}^{\mathrm{op}}, \mathbf{Cat})$ given by the functorial action of $\mathfrak{J}$ on hom-categories. One may therefore define the following.

**Definition 10.** *For any pseudofunctor* $\mathfrak{J} : \mathcal{B} \to \mathcal{X}$*, define the* extended Yoneda pseudofunctor $\underline{\mathrm{Y}} : \mathcal{B} \to \mathrm{gl}(\langle\mathfrak{J}\rangle)$ *by setting* $\underline{\mathrm{Y}}B := \big(\mathrm{Y}B, (l, \bar{l})_{(-,B)}, \mathfrak{J}B\big)$*,* $\underline{\mathrm{Y}}f := (\mathrm{Y}f, (\phi^{\mathfrak{J}}_{-,f})^{-1}, \mathfrak{J}f)$*, and* $\underline{\mathrm{Y}}(\tau : f \Rightarrow f' : B \to B') := (\mathrm{Y}\tau, \mathfrak{J}\tau)$*. The cylinder condition holds by the naturality of* $\phi^{\mathfrak{J}}$*, and the 2-cells* $\phi^{\underline{\mathrm{Y}}}$ *and* $\psi^{\underline{\mathrm{Y}}}$ *are* $(\phi^{\mathrm{Y}}, \phi^{\mathfrak{J}})$ *and* $(\psi^{\mathrm{Y}}, \psi^{\mathfrak{J}})$*, respectively.*

The extended Yoneda pseudofunctor satisfies a corresponding 'extended Yoneda lemma' (*c.f.* [15, pp. 33]).

**Lemma 5.** *For any pseudofunctor* $\mathfrak{J} : \mathcal{B} \to \mathcal{X}$ *and* $\underline{P} = (P, (\mathsf{k}, \bar{\mathsf{k}}), X) \in \mathrm{gl}(\langle\mathfrak{J}\rangle)$ *there exists an equivalence of pseudofunctors* $\mathrm{gl}(\langle\mathfrak{J}\rangle)(\underline{\mathrm{Y}}(-), \underline{P}) \simeq P$ *and an invertible modification as in the diagram below. Hence* $\underline{\mathrm{Y}}$ *is locally an equivalence.*

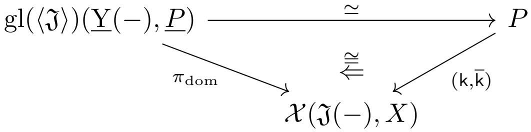

*Proof. The arrow marked* $\simeq$ *is the composite of a projection and the equivalence arising from the Yoneda lemma. Its pseudo-inverse is the composite*

$$P \xrightarrow{\simeq} \mathbf{Bicat}(\mathcal{B}^{op}, \mathbf{Cat})(\mathrm{Y}(-), P) \to \mathrm{gl}(\langle\mathfrak{J}\rangle)(\underline{\mathrm{Y}}(-), \underline{P}) \qquad (3)$$

*in which the equivalence arises from the Yoneda lemma and the unlabelled pseudofunctor takes a pseudonatural transformation* $(\mathsf{j}, \bar{\mathsf{j}}) : \mathrm{Y}B \Rightarrow P$ *to the triple with first component* $(\mathsf{j}, \bar{\mathsf{j}})$*, third component* $\mathsf{j}_B(\mathsf{k}_B(\mathrm{Id}_B)) : \mathfrak{J}B \to X$ *and second component defined using* $\bar{\mathsf{k}}$ *and* $\bar{\mathsf{j}}$*. Chasing the definitions through and evaluating at* $A, B \in \mathcal{B}$*, one sees that when* $\underline{P} := \underline{\mathrm{Y}}B$ *the composite (3) is equivalent to* $\underline{\mathrm{Y}}_{A,B}$*. Since (3) is locally an equivalence, Lemma 1(1) completes the proof.*

## 4 Cartesian closed structure on the glueing bicategory

It is well-known that, if $\mathbb{C}$ and $\mathbb{D}$ are cartesian closed categories, $\mathbb{D}$ has pullbacks, and $F : \mathbb{C} \to \mathbb{D}$ is cartesian, then $\mathrm{gl}(F)$ is cartesian closed (*e.g.* [40,12]). In this section we prove a corresponding result for the glueing bicategory. We shall be guided by the categorical proof, for which see *e.g.* [43, Proposition 2].

### 4.1 Finite products in gl($\mathfrak{J}$)

**Proposition 1.** *Let* $(\mathcal{B}, \Pi_n(-))$ *and* $(\mathcal{C}, \Pi_n(-))$ *be fp-bicategories and* $(\mathfrak{J}, \mathrm{q}^\times) : \mathcal{B} \to \mathcal{C}$ *be an fp-pseudofunctor. Then* $\mathrm{gl}(\mathfrak{J})$ *is an fp-bicategory with both projection pseudofunctors* $\pi_{\mathrm{dom}}$ *and* $\pi_{\mathrm{cod}}$ *strictly preserving products.*

For a family of objects $(C_i, c_i, B_i)_{i=1,\ldots,n}$, the $n$-ary product $\prod_{i=1}^n (C_i, c_i, B_i)$ is defined to be the tuple $\left(\prod_{i=1}^n C_i, \mathrm{q}^\times_{B_\bullet} \circ \prod_{i=1}^n c_i, \prod_{i=1}^n B_i\right)$. The $k$th projection $\underline{\pi}_k$ is $(\pi_k, \mu_k, \pi_k)$, where $\mu_k$ is defined by commutativity of the following diagram:

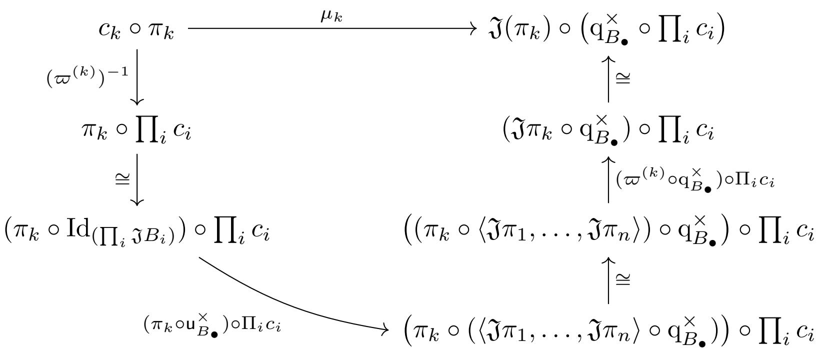

For an $n$-ary family of 1-cells $(g_i, \alpha_i, f_i) : (Y, y, X) \to (C_i, c_i, B_i)\,(i = 1, \ldots, n)$, the $n$-ary tupling is $(\langle g_1, \ldots, g_n \rangle, \{\alpha_1, \ldots, \alpha_n\}, \langle f_1, \ldots, f_n \rangle)$, where $\{\alpha_1, \ldots, \alpha_n\}$

is the composite

$$
\begin{array}{ccc}
\left(\mathrm{q}^{\times}_{B_\bullet} \circ \prod_i c_i\right) \circ \langle g_1, \dots, g_n\rangle & \xrightarrow{\{\alpha_1,\dots,\alpha_n\}} & \mathfrak{J}(\langle f_1, \dots, f_n\rangle) \circ y \\
\cong\big\downarrow & & \big\uparrow\cong \\
\mathrm{q}^{\times}_{B_\bullet} \circ \left(\prod_i c_i \circ \langle g_1, \dots, g_n\rangle\right) & & \mathrm{Id}_{\mathfrak{J}(\prod B_i)} \circ (\mathfrak{J}\langle f_1, \dots, f_n\rangle \circ y) \\
\mathrm{q}^{\times}_{B_\bullet}\circ\mathsf{fuse}\big\downarrow & & \big\uparrow(\mathsf{c}^{\times}_{B_\bullet}\circ\mathfrak{J}\langle f_1,\dots,f_n\rangle)\circ y \\
\mathrm{q}^{\times}_{B_\bullet} \circ \langle c_1 \circ g_1, \dots, c_n \circ g_n\rangle & & \left(\mathrm{q}^{\times}_{B_\bullet} \circ \langle \mathfrak{J}\pi_1, \dots, \mathfrak{J}\pi_n\rangle\right) \circ (\mathfrak{J}\langle f_1, \dots, f_n\rangle \circ y) \\
\mathrm{q}^{\times}_{B_\bullet}\circ\langle\alpha_1,\dots,\alpha_n\rangle\big\downarrow & & \big\uparrow\cong \\
\mathrm{q}^{\times}_{B_\bullet} \circ \langle \mathfrak{J}f_1 \circ y, \dots, \mathfrak{J}f_n \circ y\rangle & & \mathrm{q}^{\times}_{B_\bullet} \circ ((\langle \mathfrak{J}\pi_1, \dots, \mathfrak{J}\pi_n\rangle \circ \mathfrak{J}\langle f_1, \dots, f_n\rangle) \circ y) \\
 & & \big\uparrow\mathrm{q}^{\times}_{B_\bullet}\circ(\mathsf{unpack}^{-1}_{f_\bullet}\circ y) \\
 & \xrightarrow{\mathrm{q}^{\times}_{B_\bullet}\circ\mathsf{post}^{-1}} & \mathrm{q}^{\times}_{B_\bullet} \circ (\langle \mathfrak{J}f_1, \dots, \mathfrak{J}f_n\rangle \circ y)
\end{array}
$$

Finally, for every family of 1-cells $(g_i, \alpha_i, f_i) : (Y, y, X) \to (C_i, c_i, B_i)$ $(i = 1, \dots, n)$ we require a glued 2-cell $\underline{\pi}_k \circ (\langle g_1, \dots, g_n\rangle, \{\alpha_1, \dots, \alpha_n\}, \langle f_1, \dots, f_n\rangle) \Rightarrow (g_k, \alpha_k, f_k)$ to act as the counit. We take simply $(\varpi^{(k)}_{g_\bullet}, \varpi^{(k)}_{f_\bullet})$. This pair forms a 2-cell in $\mathrm{gl}(\mathfrak{J})$, and the required universal property holds pointwise.

*Remark 4.* If $(\mathfrak{J}, \mathrm{q}^{\times}) : \mathcal{B} \to \mathcal{X}$ is an fp-pseudofunctor, then $\underline{\mathrm{Y}} : \mathcal{B} \to \mathrm{gl}(\langle\mathfrak{J}\rangle)$ canonically extends to an fp-pseudofunctor. The pseudoinverse to $\langle \underline{\mathrm{Y}}\pi_1, \dots, \underline{\mathrm{Y}}\pi_n\rangle$ is $(\langle -, \dots, =\rangle, \cong, \mathrm{q}^{\times})$, where the component of the isomorphism at $(f_i : X \to B_i)_{i=1,\dots,n}$ is $F\langle f_\bullet\rangle \overset{\cong}{\Rightarrow} \mathrm{Id}_{F(\Pi_i B_i)} \circ F\langle f_\bullet\rangle \xRightarrow{(\mathsf{c}^{\times}_{B_\bullet})^{-1}\circ F\langle f_\bullet\rangle} \mathrm{q}^{\times}_{B_\bullet} \circ \langle F\pi_\bullet\rangle \circ F\langle f_\bullet\rangle \xRightarrow{\mathrm{q}^{\times}_{B_\bullet}\circ\mathsf{unpack}} \mathrm{q}^{\times}_{B_\bullet} \circ \langle F f_\bullet\rangle$.

## 4.2 Exponentials in gl($\mathfrak{J}$)

As in the 1-categorical case, the definition of currying in $\mathrm{gl}(\mathfrak{J})$ employs pullbacks. A *pullback* of the cospan $(X_1 \to X_0 \leftarrow X_2)$ in a bicategory $\mathcal{B}$ is a bilimit for the strict pseudofunctor $X : (1 \to 0 \leftarrow 2) \to \mathcal{B}$ determined by the cospan. We state the universal property in the form that will be most useful for our applications.

**Lemma 6.** *The pullback of a cospan* $(X_1 \xrightarrow{f_1} X_0 \xleftarrow{f_2} X_2)$ *in a bicategory* $\mathcal{B}$ *is determined, up to equivalence, by the following data and properties: a span* $(X_1 \xleftarrow{\gamma_1} P \xrightarrow{\gamma_2} X_2)$ *in* $\mathcal{B}$ *and an invertible 2-cell filling the diagram on the left below*

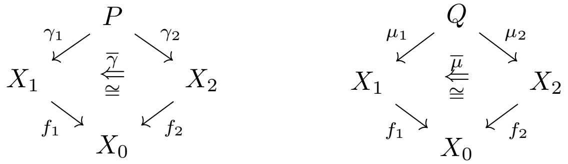

*such that*

1. *for any other diagram as on the right above there exists a* fill-in $(u, \Xi_1, \Xi_2)$, *namely a 1-cell* $u : Q \to P$ *and invertible 2-cells* $\Xi_i : \gamma_i \circ u \Rightarrow \mu_i$ $(i = 1, 2)$ *satisfying*

$$\begin{array}{ccccc}
(f_2 \circ \gamma_2) \circ u & \xrightarrow{\cong} & f_2 \circ (\gamma_2 \circ u) & \xrightarrow{f_2 \circ \Xi_2} & f_2 \circ \mu_2 \\
{\scriptstyle \overline{\gamma} \circ u} \big\downarrow & & & & \big\downarrow {\scriptstyle \overline{\mu}} \\
(f_1 \circ \gamma_1) \circ u & \xrightarrow[\cong]{} & f_1 \circ (\gamma_1 \circ u) & \xrightarrow[f_1 \circ \Xi_1]{} & f_1 \circ \mu_1
\end{array}$$

2. *for any 1-cells* $v, w : Q \to P$ *and 2-cells* $\Psi_i : \gamma_i \circ v \Rightarrow \gamma_i \circ w$ $(i = 1, 2)$ *satisfying*

$$\begin{array}{ccccccc}
(f_2 \circ \gamma_2) \circ v & \xrightarrow{\cong} & f_2 \circ (\gamma_2 \circ v) & \xrightarrow{f_2 \circ \Psi_2} & f_2 \circ (\gamma_2 \circ w) & \xrightarrow{\cong} & (f_2 \circ \gamma_2) \circ w \\
{\scriptstyle \overline{\gamma} \circ v} \big\downarrow & & & & & & \big\downarrow {\scriptstyle \overline{\gamma} \circ w} \\
(f_1 \circ \gamma_1) \circ v & \xrightarrow[\cong]{} & f_1 \circ (\gamma_1 \circ v) & \xrightarrow[f_1 \circ \Psi_1]{} & f_1 \circ (\gamma_1 \circ w) & \xrightarrow{\cong} & (f_1 \circ \gamma_1) \circ w
\end{array}$$

*there exists a unique 2-cell* $\Psi : v \Rightarrow w$ *such that* $\Psi_i = \gamma_i \circ \Psi$ $(i = 1, 2)$.

*Example 6.* 1. In **Cat**, the pullback of a cospan $(\mathcal{B} \xrightarrow{F} \mathbb{X} \xleftarrow{G} \mathcal{C})$ is the full subcategory of the comma category $(F \downarrow G)$ consisting of objects of the form $(B, f, C)$ for which $f : FB \to GC$ is an isomorphism. Note that this differs from the strict (2-)categorical pullback in **Cat**, in which every $f$ is required to be an identity (*c.f.* [65, Example 2.1]).
2. Like any bilimit, pullbacks in the bicategory $\mathbf{Bicat}(\mathcal{B}^{\mathrm{op}}, \mathbf{Cat})$ are computed pointwise (see [53, Proposition 3.6]).

We now define exponentials in the glueing bicategory. Precisely, we extend Proposition 1 to the following.

**Theorem 5.** *Let* $(\mathcal{B}, \Pi_n(-), \Rightarrow)$ *and* $(\mathcal{C}, \Pi_n(-), \Rightarrow)$ *be cc-bicategories such that* $\mathcal{C}$ *has pullbacks. For any fp-pseudofunctor* $(\mathfrak{J}, \mathrm{q}^{\times}) : (\mathcal{B}, \Pi_n(-)) \to (\mathcal{C}, \Pi_n(-))$, *the glueing bicategory* $\mathrm{gl}(\mathfrak{J})$ *has a cartesian closed structure with forgetful pseudofunctor* $\pi_{\mathrm{dom}} : \mathrm{gl}(\mathfrak{J}) \to \mathcal{B}$ *strictly preserving products and exponentials.*

*The evaluation map.* We begin by defining the mapping $(-) \Rightarrow (=)$ and the evaluation 1-cell $\underline{\mathrm{eval}}$. For $\underline{C} := (C, c, B), \underline{C'} := (C', c', B') \in \mathrm{gl}(\mathfrak{J})$ we set $\underline{C} \Rightarrow \underline{C'}$ to be the left-hand vertical leg of the following pullback diagram, in which we write $m_{B,B'} := \lambda(\mathfrak{J}(\mathrm{eval}_{B,B'}) \circ \mathrm{q}^{\times}_{B \Rightarrow B', B})$.

$$\begin{array}{ccccc}
C \supset C' & & \xrightarrow{\quad q_{c,c'} \quad} & & (C \Rightarrow C') \\
{\scriptstyle p_{c,c'}} \big\downarrow & & \overset{\omega_{c,c'}}{\Leftarrow} & & \big\downarrow {\scriptstyle \lambda(c' \circ \mathrm{eval}_{C,C'})} \\
\mathfrak{J}(B \Rightarrow B') & \xrightarrow[m_{B,B'}]{} & (\mathfrak{J}B \Rightarrow \mathfrak{J}B') & \xrightarrow[\lambda(\mathrm{eval}_{\mathfrak{J}B,\mathfrak{J}B'} \circ ((\mathfrak{J}B \Rightarrow \mathfrak{J}B') \times c))]{} & (C \Rightarrow \mathfrak{J}B')
\end{array}$$

with the lower arrow $\mathfrak{J}(B \Rightarrow B') \to (C \Rightarrow \mathfrak{J}B')$ labelled $\lambda(\mathrm{eval}_{\mathfrak{J}B,\mathfrak{J}B'} \circ ((\mathfrak{J}B \Rightarrow \mathfrak{J}B') \times c)) \circ m_{B,B'}$

(4)

*Example 7.* The pullback (4) generalises the well-known definition of a *logical relation of varying arity* [36]. Indeed, where $\mathfrak{J} := \langle\mathfrak{K}\rangle$ is the relative hom-pseudofunctor for an fp-pseudofunctor $(\mathfrak{K}, \mathrm{q}^{\times}) : \mathcal{B} \to \mathcal{X}$ between cc-bicategories, $A \in \mathcal{B}$ and $X, X' \in \mathcal{X}$, the functor $m_{X,X'}(A)$ takes a 1-cell $f : \mathfrak{K}A \to (X \Rightarrow X')$ in $\mathcal{X}$ to the pseudonatural transformation $\mathrm{Y}A \times \mathcal{X}(\mathfrak{K}(-), X) \Rightarrow \mathcal{X}(\mathfrak{K}(-), X')$ with components $\lambda B\,.\,\lambda(\rho : B \to A, u : \mathfrak{K}B \to X)\,.\,\mathrm{eval}_{X,X'} \circ \langle f \circ \mathfrak{K}(\rho), u\rangle$. Intuitively, therefore, the pullback enforces the usual closure condition defining a logical relation at exponential type, while also tracking the isomorphism witnessing that this condition holds (*c.f.* [36,3,15]).

*Notation 6.* For reasons of space—particularly in pasting diagrams—we will sometimes write $\widetilde{c} := \mathrm{eval}_{\mathfrak{J}B,\mathfrak{J}B'} \circ ((\mathfrak{J}B \Rightarrow \mathfrak{J}B') \times c) : (\mathfrak{J}B \Rightarrow \mathfrak{J}B') \times C \to \mathfrak{J}B'$ when $c : C \to \mathfrak{J}B$ in $\mathcal{C}$.

The evaluation map $\underline{\mathrm{eval}}_{\underline{C},\underline{C'}}$ is defined to be $(\mathrm{eval}_{C,C'} \circ (q_{c,c'} \times C), \mathrm{E}_{\underline{C},\underline{C'}}, \mathrm{eval}_{B,B'})$, where the witnessing 2-cell $\mathrm{E}_{\underline{C},\underline{C'}}$ is given by the pasting diagram below, in which the unlabelled arrow is $\mathrm{q}^{\times}_{(B \Rightarrow B', B)} \circ (p_{c,c'} \times c)$.

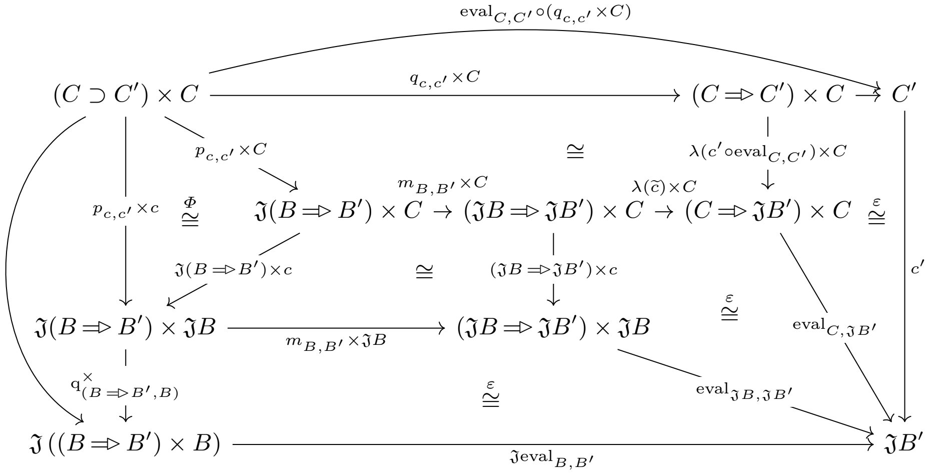

Here the bottom $\cong$ denotes a composite of $\Phi$, structural isomorphisms and $\Phi^{-1}$, and the top $\cong$ denotes a composite of $\omega_{c,c'} \times C$ with instances of $\Phi$, $\Phi^{-1}$, and the structural isomorphisms.

*The currying operation.* Let $\underline{R} := (R, r, Q)$, $\underline{C} := (C, c, B)$ and $\underline{C'} := (C', c', B')$ and suppose given a 1-cell $(t, \alpha, s) : \underline{R} \times \underline{C} \to \underline{C'}$. We construct $\underline{\lambda}(t, \alpha, s)$ using the universal property (4) of the pullback. To this end, we define invertible composites $\mathrm{U}_{\alpha}$ and $\mathrm{T}_{\alpha}$ as in the following two diagrams and set $\mathrm{L}_{\alpha} := \eta^{-1} \bullet \mathsf{e}^{\dagger}(\mathrm{U}_{\alpha}^{-1} \circ \alpha \circ \mathrm{T}_{\alpha}) : \lambda(c' \circ \mathrm{eval}_{C,C'}) \circ \lambda t \Rightarrow (\lambda(\widetilde{c}) \circ m_{B,B'}) \circ (\mathfrak{J}(\lambda s) \circ r)$.

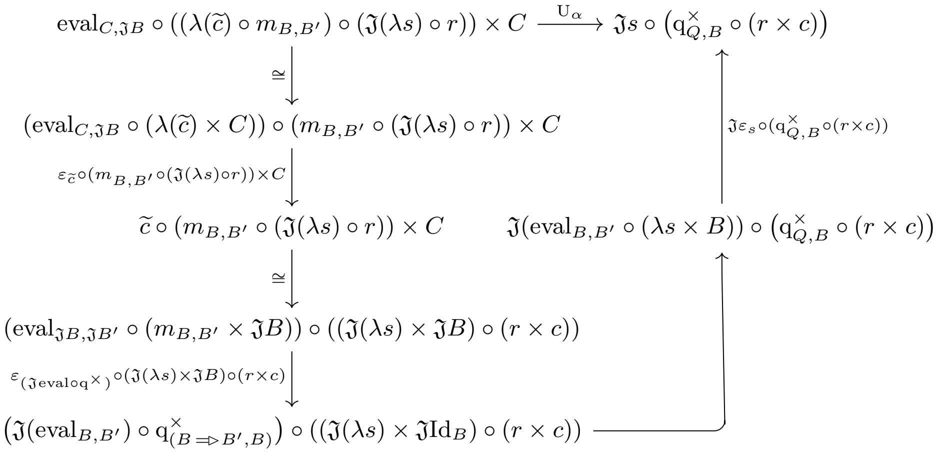

The unlabelled arrow is the canonical composite of $\mathsf{nat}_{\lambda s, \mathrm{id}_B}$ with $\phi^{\mathfrak{J}}_{\mathrm{eval}, \lambda(s) \times B}$ and structural isomorphisms. $\mathrm{T}_\alpha$ is then defined using $\mathrm{U}_\alpha$:

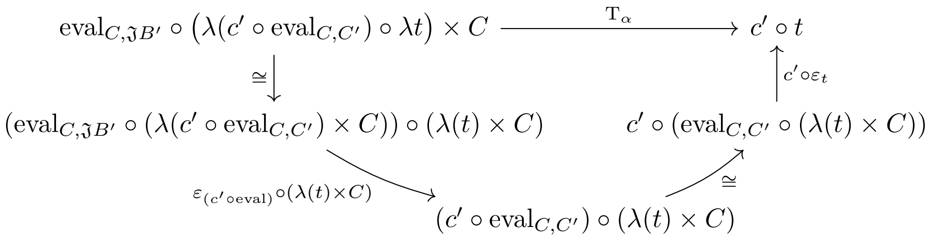

Applying the universal property of the pullback (4) to $\mathrm{L}_\alpha$, one obtains a 1-cell $\underline{\mathsf{lam}}(t)$ and a pair of invertible 2-cells $\mathit{\Gamma}_{c,c'}$ and $\mathit{\Delta}_{c,c'}$ filling the diagram

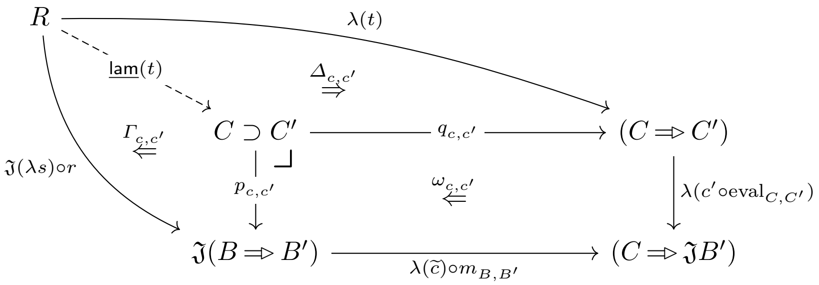

We define $\underline{\lambda}(t, \alpha, s) := \big(\underline{\mathsf{lam}}(t), \mathit{\Gamma}_{c,c'}, \lambda s\big)$.

*The counit 2-cell.* Finally we come to the counit. For a 1-cell $\underline{t} := (t, \alpha, s) : (R, r, Q) \times (C, c, B) \to (C', c', B')$ the 1-cell $\underline{\mathrm{eval}} \circ \big(\underline{\lambda}(t, \alpha, s) \times (C, c, B)\big)$ unwinds to the pasting diagram below, in which the unlabelled arrow is $\mathrm{q}^\times_{Q,B} \circ (r \times c)$:

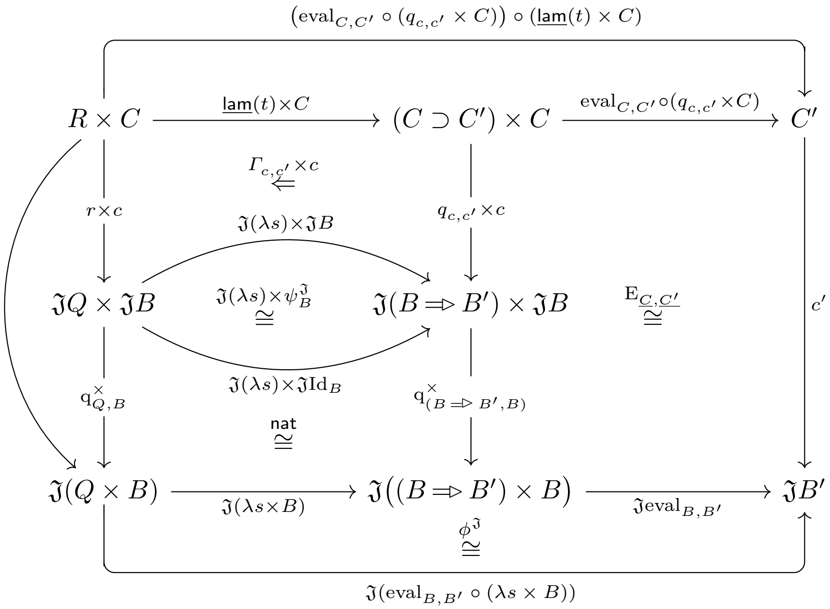

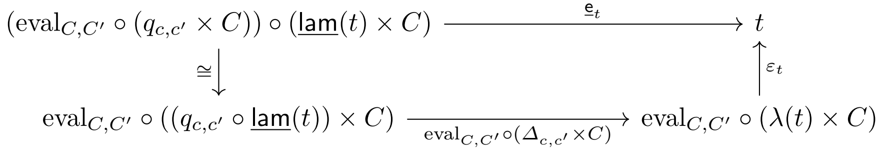

For the counit $\underline{\varepsilon_t}$ we take the 2-cell with first component $\underline{\mathsf{e}}_t$ defined by and second component simply $\varepsilon_s : \mathrm{eval}_{B,B'} \circ (\lambda(s) \times B) \Rightarrow s$. This pair forms an invertible 2-cell in $\mathrm{gl}(\mathfrak{J})$. One checks this satisfies the required universal property in a manner analogous to the 1-categorical case (see [55] for the full details). This completes the proof of Theorem 5.

## 5 Relative full completeness

We apply the theory developed in the preceding two sections to prove the relative full completeness result. As outlined in the introduction, this corresponds to a proof of *conservativity of the theory of rewriting* for the higher-order equational theory of rewriting in STLC over the algebraic equational theory of rewriting in STPC. We adapt 'Lafont's argument' [39, Annexe C] from the form presented in [16], for which we require bicategorical versions of the free cartesian category $\mathbb{F}^{\times}[\mathbb{C}]$ and free cartesian closed category $\mathbb{F}^{\times,\rightarrow}[\mathbb{C}]$ over a category $\mathbb{C}$. In line with the strategy for the STLC (*c.f.* [12, pp. 173–4]), we deal with the contravariance of the pseudofunctor $(- \Rightarrow =)$ by restricting to a bicategory of cc-pseudofunctors, pseudonatural equivalences (that is, pseudonatural transformations for which each component is a given equivalence), and invertible modifications. We denote this with the subscript $\simeq, \cong$.

**Lemma 7.** *For any bicategory $\mathcal{B}$, fp-bicategory $(\mathcal{C}, \Pi_n(-))$ and cc-bicategory $(\mathcal{D}, \Pi_n(-), \Rightarrow)$:*

1. *There exists an fp-bicategory $\mathcal{F}^{\times}[\mathcal{B}]$ and a pseudofunctor $\eta^{\times} : \mathcal{B} \to \mathcal{F}^{\times}[\mathcal{B}]$ such that composition with $\eta^{\times}$ induces a biequivalence*
$$\textbf{fp-Bicat}(\mathcal{F}^{\times}[\mathcal{B}], \mathcal{C}) \xrightarrow{\simeq} \textbf{Bicat}(\mathcal{B}, \mathcal{C})$$
2. *There exists a cc-bicategory $\mathcal{F}^{\times,\to}[\mathcal{B}]$ and a pseudofunctor $\eta^{\Rightarrow} : \mathcal{B} \to \mathcal{F}^{\times,\to}[\mathcal{B}]$ such that composition with $\eta^{\Rightarrow}$ induces a biequivalence*
$$\textbf{cc-Bicat}_{\simeq,\cong}(\mathcal{F}^{\times,\to}[\mathcal{B}], \mathcal{D}) \xrightarrow{\simeq} \textbf{Bicat}(\mathcal{B}, \mathcal{D})$$

*Proof (sketch). A syntactic construction suffices: one defines formal products and exponentials and then quotients by the axioms (see [48, p. 79] or [55]).*

Thus, for any bicategory $\mathcal{B}$, fp-bicategory $(\mathcal{C}, \Pi_n(-))$, and pseudofunctor $F : \mathcal{B} \to \mathcal{C}$ there exists an fp-pseudofunctor $F^{\#} : \mathcal{F}^{\times}[\mathcal{B}] \to \mathcal{C}$ and an equivalence $F^{\#} \circ \eta^{\times} \simeq F$. Moreover, for any fp-pseudofunctor $G : \mathcal{F}^{\times}[\mathcal{B}] \to \mathcal{C}$ such that $G \circ \eta^{\times} \simeq F$ one has $G \simeq F^{\#}$. A corresponding result holds for cc-bicategories and cc-pseudofunctors.

**Theorem 7.** *For any bicategory $\mathcal{B}$ the universal fp-pseudofunctor $\iota : \mathcal{F}^{\times}[\mathcal{B}] \to \mathcal{F}^{\times,\to}[\mathcal{B}]$ extending $\eta^{\Rightarrow}$ is locally an equivalence. Hence $\eta^{\Rightarrow} : \mathcal{B} \to \mathcal{F}^{\times,\to}[\mathcal{B}]$ is locally an equivalence.*

*Proof. Since $\iota$ preserves finite products, the bicategory $\mathrm{gl}(\langle\iota\rangle)$ is cartesian closed (Theorem 5). The composite $\mathrm{K} := \underline{\mathrm{Y}} \circ \eta^{\times} : \mathcal{B} \to \mathrm{gl}(\langle\iota\rangle)$ therefore induces a cc-pseudofunctor $\mathrm{K}^{\#} : \mathcal{F}^{\times,\to}[\mathcal{B}] \to \mathrm{gl}(\langle\iota\rangle)$.*

*First observe that $(\mathrm{K}^{\#} \circ \iota) \circ \eta^{\times} \simeq \mathrm{K}^{\#} \circ \eta^{\Rightarrow} \simeq \mathrm{K} = \underline{\mathrm{Y}} \circ \eta^{\times}$. Since $\underline{\mathrm{Y}}$ is canonically an fp-pseudofunctor (Remark 4), it follows that $\mathrm{K}^{\#} \circ \iota \simeq \underline{\mathrm{Y}}$. Since $\underline{\mathrm{Y}}$ is locally an equivalence (Lemma 5), Lemma 1(1) entails that $\mathrm{K}^{\#} \circ \iota$ is locally an equivalence.*

*Next, examining the definition of $\underline{\mathrm{Y}}$ one sees that $\pi_{\mathrm{dom}} \circ \underline{\mathrm{Y}} = \iota$, and so*
$$(\pi_{\mathrm{dom}} \circ \mathrm{K}^{\#}) \circ \eta^{\Rightarrow} \simeq (\pi_{\mathrm{dom}} \circ \underline{\mathrm{Y}}) \circ \eta^{\times} \simeq \iota \circ \eta^{\times} \simeq \eta^{\Rightarrow}$$
*It follows that $\pi_{\mathrm{dom}} \circ \mathrm{K}^{\#} \simeq \mathrm{id}_{\mathcal{F}^{\times,\to}[\mathcal{B}]}$, and hence that $\pi_{\mathrm{dom}} \circ \mathrm{K}^{\#}$ is also locally an equivalence.*

*Now consider the composite $\mathcal{F}^{\times}[\mathcal{B}] \xrightarrow{\iota} \mathcal{F}^{\times,\to}[\mathcal{B}] \xrightarrow{\mathrm{K}^{\#}} \mathrm{gl}(\langle\iota\rangle) \xrightarrow{\pi_{\mathrm{dom}}} \mathcal{F}^{\times,\to}[\mathcal{B}]$. By Lemma 1(2) and the preceding, $\iota$ is locally an equivalence. Finally, it is direct from the construction of $\mathcal{F}^{\times}[\mathcal{B}]$ that $\eta^{\times}$ is locally an equivalence; thus, so are $\iota \circ \eta^{\times} \simeq \eta^{\Rightarrow}$.*

*Acknowledgements.* We thank all the anonymous reviewers for their comments: these improved the paper substantially. We are especially grateful to the reviewer who pointed out an oversight in the original formulation of Lemma 1(2), which consequently affected the argument in Theorem 7, and provided the elegant fix therein.

The second author was supported by a Royal Society University Research Fellow Enhancement Award.

## References

[1] Abbott, M.G.: Categories of containers. Ph.D. thesis, University of Leicester (2003)

[2] Abramsky, S., Jagadeesan, R.: Games and full completeness for multiplicative linear logic. Journal of Symbolic Logic **59**(2), 543–574 (1994). 10.2307/2275407

[3] Alimohamed, M.: A characterization of lambda definability in categorical models of implicit polymorphism. Theoretical Computer Science **146**(1-2), 5–23 (1995). 10.1016/0304-3975(94)00283-O

[4] Balat, V., Di Cosmo, R., Fiore, M.: Extensional normalisation and typed-directed partial evaluation for typed lambda calculus with sums. In: Proceedings of the 31st Annual ACM SIGPLAN-SIGACT Symposium on Principles of Programming Languages. pp. 64–76 (2004)

[5] Bénabou, J.: Introduction to bicategories. In: Reports of the Midwest Category Seminar. pp. 1–77. Springer Berlin Heidelberg, Berlin, Heidelberg (1967)

[6] Bloom, S.L., Ésik, Z., Labella, A., Manes, E.G.: Iteration 2-theories. Applied Categorical Structures **9**(2), 173–216 (2001). 10.1023/a:1008708924144

[7] Borceux, F.: Bicategories and distributors, Encyclopedia of Mathematics and its Applications, vol. 1, pp. 281–324. Cambridge University Press (1994). 10.1017/CBO9780511525858.009

[8] Carboni, A., Kelly, G.M., Walters, R.F.C., Wood, R.J.: Cartesian bicategories II. Theory and Applications of Categories **19**(6), 93–124 (2008), http://www.tac.mta.ca/tac/volumes/19/6/19-06abs.html

[9] Carboni, A., Lack, S., Walters, R.F.C.: Introduction to extensive and distributive categories. Journal of Pure and Applied Algebra **84**(2), 145–158 (1993). 10.1016/0022-4049(93)90035-r

[10] Carboni, A., Walters, R.F.C.: Cartesian bicategories I. Journal of Pure and Applied Algebra **49**(1), 11–32 (1987). 10.1016/0022-4049(87)90121-6

[11] Castellan, S., Clairambault, P., Rideau, S., Winskel, G.: Games and strategies as event structures. Logical Methods in Computer Science **13** (2017)

[12] Crole, R.L.: Categories for Types. Cambridge University Press (1994). 10.1017/CBO9781139172707

[13] Dagand, P.E., McBride, C.: A categorical treatment of ornaments. In: Proceedings of the 28th Annual ACM/IEEE Symposium on Logic in Computer Science. pp. 530–539. IEEE Computer Society, Washington, DC, USA (2013). 10.1109/LICS.2013.60

[14] Fiore, M.: Axiomatic Domain Theory in Categories of Partial Maps. Distinguished Dissertations in Computer Science, Cambridge University Press (1996)

[15] Fiore, M.: Semantic analysis of normalisation by evaluation for typed lambda calculus. In: Proceedings of the 4th ACM SIGPLAN International Conference on Principles and Practice of Declarative Programming. pp. 26–37. ACM, New York, NY, USA (2002). 10.1145/571157.571161

[16] Fiore, M., Di Cosmo, R., Balat, V.: Remarks on isomorphisms in typed lambda calculi with empty and sum types. In: Proceedings of the 28th Annual IEEE Symposium on Logic in Computer Science. pp. 147–156. IEEE Computer Society Press (2002). 10.1109/LICS.2002.1029824

[17] Fiore, M., Gambino, N., Hyland, M., Winskel, G.: The cartesian closed bicategory of generalised species of structures. Journal of the London Mathematical Society **77**(1), 203–220 (2007). 10.1112/jlms/jdm096

[18] Fiore, M., Gambino, N., Hyland, M., Winskel, G.: Relative pseudomonads, Kleisli bicategories, and substitution monoidal structures. Selecta Mathematica New Series (2017)

[19] Fiore, M., Joyal, A.: Theory of para-toposes. Talk at the Category Theory 2015 Conference. Departamento de Matematica, Universidade de Aveiro (Portugal)

[20] Fiore, M., Saville, P.: A type theory for cartesian closed bicategories. In: Proceedings of the 34th Annual ACM/IEEE Symposium on Logic in Computer Science (2019). 10.1109/LICS.2019.8785708

[21] Fiore, M., Saville, P.: Coherence and normalisation-by-evaluation for bicategorical cartesian closed structure. Preprint (2020)

[22] Fiore, M., Simpson, A.: Lambda definability with sums via Grothendieck logical relations. In: Girard, J.Y. (ed.) Typed lambda calculi and applications: 4th international conference. pp. 147–161. Springer Berlin Heidelberg, Berlin, Heidelberg (1999)

[23] Freyd, P.: Algebraically complete categories. In: Lecture Notes in Mathematics, pp. 95–104. Springer Berlin Heidelberg (1991). 10.1007/bfb0084215

[24] Freyd, P.J., Scedrov, A.: Categories, Allegories. Elsevier North Holland (1990)

[25] Gambino, N., Joyal, A.: On operads, bimodules and analytic functors. Memoirs of the American Mathematical Society **249**(1184), 153–192 (2017)

[26] Gambino, N., Kock, J.: Polynomial functors and polynomial monads. Mathematical Proceedings of the Cambridge Philosophical Society **154**(1), 153–192 (2013). 10.1017/S0305004112000394

[27] Ghani, N.: Adjoint rewriting. Ph.D. thesis, University of Edinburgh (1995)

[28] Gibbons, J.: Conditionals in distributive categories. Tech. rep., University of Oxford (1997)

[29] G.L. Cattani, Fiore, M., Winskel, G.: A theory of recursive domains with applications to concurrency. In: Proceedings of the 13th Annual IEEE Symposium on Logic in Computer Science. pp. 214–225. IEEE Computer Society (1998)

[30] Gurski, N.: An Algebraic Theory of Tricategories. University of Chicago, Department of Mathematics (2006)

[31] Hasegawa, M.: Logical predicates for intuitionistic linear type theories. In: Girard, J.Y. (ed.) Typed lambda calculi and applications: 4th international conference. pp. 198–213. Springer Berlin Heidelberg, Berlin, Heidelberg (1999)

[32] Hilken, B.: Towards a proof theory of rewriting: the simply typed 2$$\lambda $$-calculus. Theoretical Computer Science **170**(1), 407–444 (1996). 10.1016/S0304-3975(96)80713-4

[33] Hirschowitz, T.: Cartesian closed 2-categories and permutation equivalence in higher-order rewriting. Logical Methods in Computer Science **9**, 1–22 (2013)

[34] Jay, C.B., Ghani, N.: The virtues of eta-expansion. Journal of Functional Programming **5**(2), 135–154 (1995). 10.1017/S0956796800001301

[35] Johann, P., Polonsky, P.: Higher-kinded data types: Syntax and semantics. In: 34th Annual ACM/IEEE Symposium on Logic in Computer Science. IEEE (2019). 10.1109/lics.2019.8785657

[36] Jung, A., Tiuryn, J.: A new characterization of lambda definability. In: Bezem, M., Groote, J.F. (eds.) Typed Lambda Calculi and Applications. pp. 245–257. Springer Berlin Heidelberg, Berlin, Heidelberg (1993)

[37] Lack, S.: A 2-Categories Companion, pp. 105–191. Springer New York, New York, NY (2010)

[38] Lack, S., Walters, R.F.C., Wood, R.J.: Bicategories of spans as cartesian bicategories. Theory and Applications of Categories **24**(1), 1–24(2010)

[39] Lafont, Y.: Logiques, catégories et machines. Ph.D. thesis, UniversitéParis VII (1987)

[40] Lambek, J., Scott, P.J.: Introduction to Higher Order Categorical Logic. Cambridge University Press, New York, NY, USA (1986)

[41] Leinster, T.: Basic bicategories (May 1998), https://arxiv.org/pdf/math/9810017.pdf

[42] Leinster, T.: Higher operads, higher categories. No. 298 in London Mathematical Society Lecture Note Series, Cambridge University Press (2004)

[43] Ma, Q.M., Reynolds, J.C.: Types, abstraction, and parametric polymorphism, part 2. In: Brookes, S., Main, M., Melton, A., Mislove, M., Schmidt, D. (eds.) Mathematical Foundations of Programming Semantics. pp. 1–40. Springer Berlin Heidelberg, Berlin, Heidelberg (1992)

[44] Mac Lane, S.: Categories for the Working Mathematician, Graduate Texts in Mathematics, vol. 5. Springer-Verlag New York, second edn. (1998). 10.1007/978-1-4757-4721-8

[45] Mac Lane, S., Paré, R.: Coherence for bicategories and indexed categories. Journal of Pure and Applied Algebra **37**, 59–80 (1985). 10.1016/0022-4049(85)90087-8

[46] Marmolejo, F., Wood, R.J.: Kan extensions and lax idempotent pseudomonads. Theory and Applications of Categories **26**(1), 1–29 (2012)

[47] Mitchell, J.C., Scedrov, A.: Notes on sconing and relators. In: Börger, E.,J., G., Kleine Büning, H., Martini, S., Richter, M.M. (eds.) Computer Science Logic. pp. 352–378. Springer Berlin Heidelberg, Berlin, Heidelberg (1993)

[48] Ouaknine, J.: A two-dimensional extension of Lambek’s categorical proof theory. Master’s thesis, McGill University (1997)

[49] Paquet, H.: Probabilistic concurrent game semantics. Ph.D. thesis, University of Cambridge (2020)

[50] Plotkin, G.D.: Lambda-definability and logical relations. Tech. rep., University of Edinburgh School of Artificial Intelligence (1973), memorandum SAI-RM-4

[51] Power, A.J.: An abstract formulation for rewrite systems. In: Pitt, D.H., Rydeheard, D.E., Dybjer, P., Pitts, A.M., Poigné, A. (eds.) Category Theory and Computer Science. pp. 300–312. Springer Berlin Heidelberg, Berlin, Heidelberg (1989)

[52] Power, A.J.: Coherence for bicategories with finite bilimits I. In: Gray, J.W., Scedrov, A. (eds.) Categories in Computer Science and Logic: Proceedings of the AMS-IMS-SIAM Joint Summer Research Conference, vol. 92, pp. 341–349. AMS (1989)

[53] Power, A.J.: A general coherence result. Journal of Pure and Applied Algebra **57**(2), 165–173 (1989). 10.1016/0022-4049(89)90113-8

[54] Rydeheard, D.E., Stell, J.G.: Foundations of equational deduction: A categorical treatment of equational proofs and unification algorithms. In: Pitt, D.H., Poigné, A., Rydeheard, D.E. (eds.) Category Theory and Computer Science. pp. 114–139. Springer Berlin Heidelberg, Berlin, Heidelberg (1987)

[55] Saville, P.: Cartesian closed bicategories: type theory and coherence. Ph.D. thesis, University of Cambridge (Submitted)

[56] Seely, R.A.G.: Modelling computations: A 2-categorical framework. In: Gries, D.(ed.) Proceedings of the 2nd Annual IEEE Symposium on Logic in Computer Science. pp. 65–71. IEEE Computer Society Press (June 1987)

[57] Statman, R.: Logical relations and the typed $$\lambda $$-calculus. Information and Control **65**, 85–97 (1985)

[58] Stell, J.: Modelling term rewriting systems by sesqui-categories. In: Proc. Catégories, Algèbres, Esquisses et Néo-Esquisses (1994)

[59] Street, R.: Fibrations in bicategories. Cahiers de Topologie et Géométrie Différentielle Catégoriques **21**(2), 111–160 (1980), https://eudml.org/doc/91227

[60] Street, R.: Categorical structures. In: Hazewinkel, M. (ed.) Handbook of Algebra, vol. 1, chap. 15, pp. 529–577. Elsevier (1995)

[61] Tabareau, N.: Aspect oriented programming: A language for 2-categories. In: Proceedings of the 10th International Workshop on Foundations of Aspect-oriented Languages. pp. 13–17. ACM, New York, NY, USA (2011).10.1145/1960510.1960514

[62] Taylor, P.: Practical Foundations of Mathematics, Cambridge Studies in Advanced Mathematics, vol. 59. Cambridge University Press (1999)

[63] Troelstra, A.S., Schwichtenberg, H.: Basic proof theory. No. 43 in Cambridge Tracts in Theoretical Computer Science, Cambridge University Press, second edn. (2000)

[64] Verity, D.: Enriched categories, internal categories and change of base. Ph.D. thesis, University of Cambridge (1992), TAC reprint available at http://www.tac.mta.ca/tac/reprints/articles/20/tr20abs.html

[65] Weber, M.: Yoneda structures from 2-toposes. Applied Categorical Structures **15**(3), 259–323 (2007). 10.1007/s10485-007-9079-2

